# The first complete mitogenome of the South China deep‐sea giant isopod *Bathynomus* sp. (Crustacea: Isopoda: Cirolanidae) allows insights into the early mitogenomic evolution of isopods

**DOI:** 10.1002/ece3.2737

**Published:** 2017-02-16

**Authors:** Yanjun Shen, Qi Kou, Zaixuan Zhong, Xinzheng Li, Lisheng He, Shunping He, Xiaoni Gan

**Affiliations:** ^1^ The Key Laboratory of Aquatic Biodiversity and Conservation of Chinese Academy of SciencesInstitute of HydrobiologyChinese Academy of SciencesWuhanHubeiChina; ^2^ University of Chinese Academy of SciencesBeijingChina; ^3^ Institute of OceanologyChinese Academy of SciencesQingdaoChina; ^4^ Institute of Deep‐sea Science and EngineeringChinese Academy of SciencesSanyaChina

**Keywords:** *Bathynomus*, deep sea, gene rearrangement, Isopoda, mitochondrial genome

## Abstract

In this study, the complete mitochondrial (mt) genome sequence of the South China deep‐sea giant isopod *Bathynomus* sp. was determined, and this study is the first to explore in detail the mt genome of a deep‐sea member of the order Isopoda. This species belongs to the genus *Bathynomus*, the members of which are saprophagous residents of the deep‐sea benthic environment; based on their large size, *Bathynomus* is included in the “supergiant group” of isopods. The mt genome of *Bathynomus* sp. is 14,965 bp in length and consists of 13 protein‐coding genes, two ribosomal RNA genes, only 18 transfer RNA genes, and a noncoding control region 362 bp in length, which is the smallest control region discovered in Isopoda to date. Although the overall genome organization is typical for metazoans, the mt genome of *Bathynomus* sp. shows a number of derived characters, such as an inversion of 10 genes when compared to the pancrustacean ground pattern. Rearrangements in some genes (e.g., *cob*,* trnT*,* nad5,* and *trnF*) are shared by nearly all isopod mt genomes analyzed thus far, and when compared to the putative isopod ground pattern, five rearrangements were found in *Bathynomus* sp. Two tRNAs exhibit modified secondary structures: The TΨC arm is absent from *trnQ*, and *trnC* lacks the DHU. Within the class Malacostraca, *trnC* arm loss is only found in other isopods. Phylogenetic analysis revealed that *Bathynomus* sp. (Cymothoida) and *Sphaeroma serratum* (Sphaeromatidea) form a single clade, although it is unclear whether Cymothoida is monophyletic or paraphyletic. Moreover, the evolutionary rate of *Bathynomus* sp. (dN/dS [nonsynonymous mutational rate/synonymous mutational rate] = 0.0705) is the slowest measured to date among Cymothoida, which may be associated with its relatively constant deep‐sea environment. Overall, our results may provide useful information for understanding the evolution of deep‐sea Isopoda species.

## 1 Introduction

Mitochondria, which are regarded as relicts of bacterial or alpha‐bacterial endosymbionts incorporated into an early eukaryotic cell, have their own genetic material and genetic system. Since the discovery of intraorganellar DNA in chick embryo mitochondria (Nass & Nass, 1963), determination of the structure of mitochondrial genomes (mitogenomes) has become an important aspect of genome research because of the important data it can provide for phylogenetic analyses. In general, the metazoan mitogenome is a circular, double‐stranded DNA molecule approximately 12–20 kb in length that consists of the same 37 genes: 13 protein‐coding subunits, two ribosomal RNAs, and 22 transfer RNAs (Wolstenholme, 1992). Due to the different rates of evolutionary change among different segments of a given mitogenome (Helfenbein, Fourcade, Vanjani, & Boore, 2004; Liebers, de Knijff, & Helbig, 2004), the gene order (Roehrdanz, Degrugillier, & Black, 2002), and the RNA secondary structure (Macey, Schulte, & Larson, 2000), these sequences can provide large datasets for phylogenetic analyses at different levels and can also serve as ideal models of gene rearrangement and genome evolution.

Since Clary and Wolstenholme (1985) sequenced the mitogenome of *Drosophila yakuba* in 1985, 1,160 Arthropoda mitogenomes have been determined. However, only 203 mitogenomes within the class Crustacea have been determined, and the number of isopod mitogenomes currently available is even lower, with only three complete mitogenomes (one each for Limnoriidae, Ligiidae, and Phreatoicidae) and another eight sequences that are almost complete (one each for Sphaeromatidae, Chaetiliidae, Cirolanidae, Asellidae, Armadillidiidae, Idoteidae, Cylisticidaeand, and Trachelipodidae; see Table 1). Among crustacean mitogenomes, most share the ancestral pancrustacean (crustacean + hexapod) gene order that shows only a *trnL‐UUR* translocation relative to the ancestral arthropod arrangement found in the horseshoe crab *Limulus polyphemus* (Lavrov, Boore, & Brown, 2000) or present only minor tRNA translocations (Yang & Yang, 2008). Nonetheless, a broad comparison of mitochondrial (mt) gene order within Crustacea has revealed that some taxa exhibit greater variability, e.g., Copepoda, Cirripedia, Brachyura, and Isopoda (Kilpert & Podsiadlowski, 2006).

**Table 1:** All isopod mitogenomes sequenced to date and their nucleotide compositions

Species	Suborder/infraorder	Accession number	Length (bp)	Entire genome			Protein‐coding gene			rrnL		rrnS		tRNAs		Control region		Reference
				AT%	GC skew	AT skew	Length (aa)	AT% (all)	AT% (3rd)	Length (bp)	AT%	Length (bp)	AT%	Length (bp)/number	AT%	Length (bp)	AT%	
*Bathynomus* sp.^a^	Cymothoida	KU057374	14,965	58.7	+0.177	−0.094	3,713	57.7	59.7	1,181	60.5	742	59.7	1,080/18	63.6	362	60.2	This study
*Limnoria quadripunctata*	Cymothoida	KF704000	16,515	66.4	+0.190	−0.102	3,692	64.8	67.4	1,367	71.6	740	71.8	1,500/24	72.6	1,525	66.0	Lloyd et al. (2015)
*Ligia oceanica*	Oniscidea	DQ442914	15,289	60.9	+0.133	−0.041	3,677	60.1	64.4	1,234	65.1	850	60.2	1,278/21	65.0	737	55.8	Kilpert et al. (2006)
*Eophreatoicus* sp. ‐14	Phreatoicidae	NC_013976	14,994	69.6	+0.250	−0.103	3,687	69.1	79.0	1,224	73.2	784	71.7	1,376/22	71.8	401	58.6	Kilpert et al. (2010)
*Sphaeroma serratum* b	Sphaeromatidea	GU130256	13,467	54.4	+0.022	−0.070	3,567	53.6	50.4	1,154	62.3	651	43.1	917/15	62.8	–	–	Kilpert et al. (2012)
*Glyptonotus cf. Antarcticus* b	Valvifera	GU130254	13,809	65.4	+0.040	−0.034	3,567	64.6	71.6	1,215	72.7	745	62.2	1,117/18	67.9	–	–	Kilpert et al. (2012)
*Eurydice pulchra* b	Cymothoida	GU130253	13,055	55.9	+0.197	−0.052	3,386	55.8	61.4	1,137	59.0	698	50.5	964/16	57.3	–	–	Kilpert et al. (2012)
*Asellus aquaticus* b	Asellota	GU130252	13,639	61.9	−0.121	+0.002	3,561	60.3	66	1,168	65.9	675	69.5	1,099/18	69.3	–	–	Kilpert et al. (2012)
*Armadillidium vulgare* c	Oniscidea	EF643519	13,939	71.2	+0.181	−0.042	3,746	70.8	82.2	600	71.7	687	68.0	715/12	75.2	–	–	Marcade et al. (2007)
*Idotea baltica* c	Valvifera	DQ442915	14,247	61.0	+0.164	−0.075	3,627	60.2	64.5	1,216	65.6	794	57.8	1,051/17	65.2	–	–	Podsiadlowski et al. (2006)
*Cylisticus convexus* c	Oniscidea	KR013002	14,154	67.8	+0.196	−0.035	3,668	67.5	75.0	979	72.8	812	60.2	1,106/18	68.9	–	–	Chandler et al. (2015)
*Trachelipus rathkei* c	Oniscidea	KR013001	14,129	67.4	+0.184	−0.030	3,625	66.5	74.0	975	71.9	807	64.1	846/14	69.4	–	–	Chandler et al. (2015)

a Results for *Bathynomus* sp. from this study are given in bold.

b Incomplete determination with a partial *cob* sequence, and a few tRNAs and the control region were not sequenced.

c Incomplete determination, with a few tRNAs and the control region not sequenced.

Approximately 77% of the ocean floor and 60% of our planet's surface are covered with deep‐sea habitats that remain largely unexplored. In these deep‐sea environments, a lack of sunlight, extremely high pressures, and low oxygen levels prevent the formation of typical biological assemblages due to the lack of photosynthesis, which is responsible for biosphere primary production. Nonetheless, the deep sea is a species‐rich habitat, as has been well documented since marine biologists began to extensively sample the bathyal and abyssal depths (Grassle, 1989; Hessler & Sanders, 1967; Wolff, 1977). For example, Isopoda comprises a highly diverse and species‐rich group of crustaceans, with many members living in the abyssal benthos in all oceans (Hessler, Wilson, & Thistle, 1979; Wolff, 1962), and the existence of such biological communities has produced a profound change in our perception of deep‐sea life (Van Dover, German, Speer, Parson, & Vrijenhoek, 2002). In addition, mitochondria are the energy metabolism centers of the cell because more than 95% of cellular energy is generated by mitochondria through oxidative phosphorylation (OXPHOS). Mitochondrial‐encoded OXPHOS genes may therefore evolve under selection due to metabolic requirements and display evidence of adaptive evolution in mammals, birds, and fishes (Shen, Shi, Sun, & Zhang, 2009; Sun, Shen, Irwin, & Zhang, 2011).

Because of their large size, *Bathynomus* spp. (Crustacea: Isopoda: Cirolanidae) are classified into the “supergiant group” of isopods (Lowry & Dempsey, 2006). These animals are important scavengers in the deep‐sea benthic environment, from the gloomy sublittoral zone, at a depth of 170 m, to the dark of the bathypelagic zone at 2,140 m, and they are often found at depths between 365 and 730 m (Holthuis & Mikulka, 1972). Sankar et al. (2011) also mentioned these species as the first recorded deep‐sea isopods in the waters off India. However, no complete mitogenome data are available to date for any deep‐sea isopod, even though studying the early mitogenomic evolution of deep‐sea isopods using mt DNA fragments is facilitated by effective Crustacea‐specific versatile primers (Crandall & Fitzpatrick, 1996; Folmer, Black, Hoeh, Lutz, & Vrijenhoek, 1994; Merritt et al., 1998).

In this study, we report the first complete mitogenome sequence of the *Bathynomus* sp. mitogenome, consisting of the same 13 protein‐coding genes and two rRNAs found in most metazoans but only containing 18 tRNAs, with *tRNA‐H*,* tRNA‐I*,* tRNA‐L1,* and *tRNA‐S1* missing from the typical structure. Moreover, the genes show surprising rearrangements compared to the ancestral pancrustacean order and the isopod ground pattern. Phylogenetic analyses indicate that *Bathynomus* sp. (Cymothoida) and *Sphaeroma serratum* (Sphaeromatidea) form one clade and that the evolutionary rate of *Bathynomus* sp. (dN/dS = 0.0705) is the slowest measured to date within Cymothoida. Our results may provide useful information for understanding the evolution of deep‐sea isopod species.

## 2 Materials and Methods

Experiments were performed in accordance with the recommendations of the Ethics Committee of the Institute of Hydrobiology, Chinese Academy of Sciences. These policies were enacted according to the Chinese Association for Laboratory Animal Sciences and the Institutional Animal Care and Use Committee protocols.

### 2.1 Sampling, identification, and DNA extraction

Six individuals of *Bathynomus* sp. were collected at a depth of 898 m in the South China sea (110°38.217′E, 17°46.845′N) using the “ROV Lander 01,” which was a remotely operated vehicle (ROV) with sampling equipment made by the Sanya Institute of Deep‐sea Science and Engineering, Chinese Academy of Sciences, in November 2014 (Fig. S1). All specimens were preserved in 99% ethanol until DNA extraction. Species‐level morphological identification was performed based on original morphological descriptions, locality data, and additional information about the giant deep‐sea scavenger genus *Bathynomus* (Lowry & Dempsey, 2006). Total genomic DNA was extracted from one or several pleopods using the EZNA^®^ Tissue DNA Kit (OMEGA, Wuhan, China) following the manufacturer's instructions.

### 2.2 Genome determination

The first partial genetic fragments of the genes encoding cytochrome oxidase subunit 1 (*cox1*), cytochrome oxidase subunit 2 (*cox2*), large ribosomal RNA (*rrnL*), small ribosomal RNA (*rrnS*), cytochrome b (*cob*), and NADH dehydrogenase subunit 5 (*nad5*) were amplified using primers LCO1490/HCO2198 (Folmer et al., 1994), CO2f/CO2r1 (Yamauchi, Miya, & Nishida, 2004), 16sf‐cray/16s1472 (Crandall & Fitzpatrick, 1996), SR‐J14197/SR‐N14745 (Simon, Buckley, Frati, Stewart, & Beckenbach, 2006), Cytb151F/Cytb270R (Merritt et al., 1998), and DEnad5F/DEnad5R (Yang & Yang, 2008), respectively (Table S1). The reactions were carried out in a volume of 30 μl containing 21.125 μl of sterilized ultrapure water, 3.0 μl of 10× PCR buffer (including MgCl_2_), 1.5 μl of each primer (10 mmol/L), 1.5 μl of dNTPs (2.5 mmol/L each), 0.375 μl of Taq DNA polymerase (2.5 U/μl, Takara Bio, Shanghai, China), and 1.0 μl of DNA template (50–100 ng/μl). The cycling parameters were as follows: 94°C for 5 min; 32 cycles of 94°C for 40 s, 43–48°C for 30 s, and 72°C for 1 min; and a final elongation step at 72°C for 10 min. The six amplified fragments were sequenced and used for designing gene‐specific primers.

To facilitate the subsequent long PCR, we chose candidates with high melting temperatures (Table S1). Long PCRs were performed in a volume of 25 μl containing 12.75 μl of sterilized ultrapure water, 2.5 μl of 10× Takara LA PCR buffer (including MgCl_2_), 4 μl of Takara dNTP mixture (2.5 mmol/L each), 0.25 μl of Takara LA Taq polymerase (5 units/μl), 1 μl of primer mixture (10 mmol/L each), and 4 μl of DNA template (100 ng/μl). The corresponding thermal cycler protocol consisted of an initial denaturation step (94°C, 1 min), followed by 30 cycles of denaturation (98°C, 10 s), annealing and extension (64–68°C, 12 min), and ending with another extension (72°C, 10 min). The long PCR products were sequenced using a primer‐walking strategy. Considering the lack of data regarding genome arrangement for this species and to obtain precise genomic sequences, five gene‐specific primer pairs were designed to cover any gaps and inaccurate sequencing.

All of the PCR products were visualized on 1.2% low‐melting agarose gels stained with ethidium bromide. The products were then purified and sequenced using an ABI3730XL sequencing system, and the primers are described in Table S1.

### 2.3 Gene annotation and sequence analysis

Overlapping fragments obtained by sequencing were edited and aligned using BioEdit v7.0.9.0 (Hall, 1999). The MITOS webserver (Bernt et al., 2013) was used to annotate the *Bathynomus* sp. mitogenome, and protein‐coding and ribosomal RNA genes were rechecked by aligning them with publicly available mitogenomes of 11 isopod species (Table 1). The reliable identification of tRNA genes is generally not trivial, and the most common approach is to search for base pairings that conform to a typical tRNA clover‐leaf structure. Therefore, tRNAs were initially re‐detected using two computer programs: tRNAscan‐SE version 1.21 (Lowe & Eddy, 1997) and ARWEN 1.2.3.c (Laslett & Canback, 2008). Confirmation was performed by blast searches of all tRNAs in MitoZoa 2.0 (de Meo et al., 2012).

CGView (Stothard & Wishart, 2005) was used for a circular display of the *Bathynomus* sp. mitogenome, which was modified manually. The complete genome sequence has been submitted to NCBI (GenBank: KU057374). The nucleotide composition was analyzed with MEGA 5 (Tamura et al., 2011). Strand skew values were calculated according to the formulae given by Perna and Kocher (1995): AT skew = (A − T)/(A + T) and GC skew = (G − C)/(G + C), where A, T, C, G are the four bases.

### 2.4 Phylogenetic analysis and evolutionary rate estimation

We utilized the 11 other determined isopod mitogenomes for phylogenetic analyses (Table 1); six decapod species were used as outgroups: *Alvinocaris longirostris* (AB821296), *Austinograea rodriguezensis* (JQ035658), *Geothelphusa dehaani* (AB187570), *Halocaridina rubra strain* (KF437508), *Panulirus japonicus* (NC_004251), and *Shinkaia crosnieri* (NC_011013). Both the nucleotides and amino acids of the 13 protein‐coding genes were subjected to concatenated alignments using MEGA 5 (Tamura et al., 2011). Poorly aligned positions and divergent regions were removed using Gblocks version 0.91b (Castresana, 2000) set at the default block parameters. The final nucleotide and amino acid datasets consisted of 10,071 nt and 3,313 aa, respectively.

For the nucleotide dataset, jModelTest 2 (Darriba, Taboada, Doallo, & Posada, 2012) was used to select the model GTR + I + G for both maximum likelihood (ML) and Bayesian analyses. ProtTest version 3.4 (Darriba, Taboada, Doallo, & Posada, 2011) was used to choose the model MtArt + G + F for the amino acid dataset. As MtArt could not be implemented in the amino acid Bayesian analysis, we used the best scoring alternative MtRev + I + G + F model.

Maximum likelihood analyses of the nucleotide and amino acid alignments were assembled in PhyML 3.0 (Guindon & Gascuel, 2003), with 1,000 replicates performed under the models GTR + G + I and MtArt + G + F, respectively. Bayesian analyses of the nucleotide and amino acid alignments were carried out using MrBayes 3.1.2 (Ronquist & Huelsenbeck, 2003) under the models GTR + G + I and MtRev + I + G + F (see above), respectively, with 2,000,000 generations in two runs of eight chains each.

To estimate the evolutionary rate, standard branch models calculated with the CODEML program of PAML 4.6 (Yang, 2007) were used, and an adjusted Chi‐square test (Storey & Tibshirani, 2003) was applied for testing *p* values.

## 3 Results

### 3.1 Mitogenome organization

**Figure 1:**
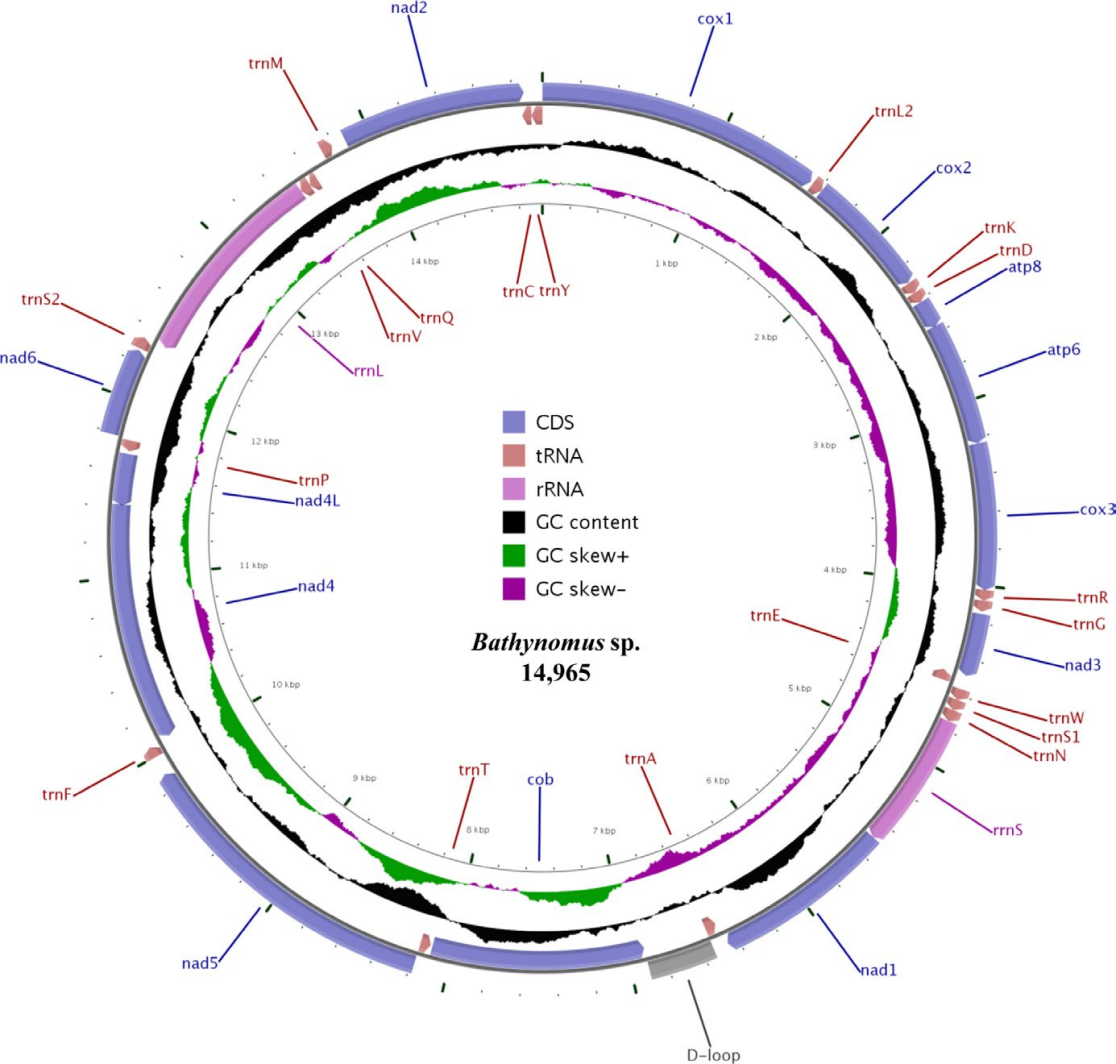
Map of the *Bathynomus* sp. mitogenome. Transfer RNAs are represented by their one‐letter amino acid code. Arrows pointing clockwise indicate (+) strand genes; counterclockwise arrows indicate (−) strand genes. The figure was initially generated by CGView and then modified manually

The mitogenome of the South China deep‐sea giant isopod *Bathynomus* sp. is a 14,965‐bp circular molecule (Figure 1) with a nucleotide composition of A% = 26.6, G% = 24.3, T% = 32.1, and C% = 17.0. The genome has an overall AT content of 58.7%, which appears to be low for isopods (typical range: 54.4%–71.2%). The composition is skewed away from G in favor of C (the GC skew is +0.177) but is almost balanced for A and T (the AT skew is −0.094). This feature is well conserved among isopods (Table 1).

This species has the smallest complete mitogenome found in isopods (Table 1) thus far. The genome contains the same 13 protein‐coding genes and two ribosomal RNAs found in most metazoans. However, it exhibits incomplete tRNA‐encoding capacity (18 tRNAs instead of the usual 22, see Table 2). Twenty‐one mt genes are transcribed from one strand (the plus strand) and the remaining 12 from the other (the minus strand). A total of 817 noncoding intergenic nucleotides were found, with the largest continuous region (362 bp, AT% = 60.2) located between *trnA* and *cob*. Due to its location and AT richness, we predict that this part of the genome is the mt control region and that it likely contains the origins of replication and regulatory elements for transcription. However, this predicted control region is considerably smaller than that in other isopods (Table 1) and shows no sequence similarity with any previously sequenced Isopoda control region (data not shown).

**Table 2:** Gene content of the *Bathynomus* sp. mitogenome

Gene	Strand^b^	GenBank position no.	Size (nts)	Start codon	Stop codon	Anticodon	Intergenic nucleotides
cox1	+	1–1,539	1,539	ATG	TAG		14
trnL2‐UUR	+	1,554–1,614	61			TAA	0
cox2	+	1,615–2,292	678	ATT	TAA		3
trnK	+	2,296–2,355	60			TTT	0
trnD	+	2,356–2,415	60			GTC	0
atp8	+	2,416–2,568	153	ATT	TGA		−4^c^
atp6	+	2,565–3,233	669	ATG	TAA		0
cox3	+	3,234–4,022	789	ATG	TAA		−2
trnR	+	4,021–4,078	58			TCG	0
trnG	+	4,079–4,138	60			TCC	12
nad3	+	4,151–4,492	342	ATT	TAG		0
trnE	−	4,493–4,551	59			TTC	16
trnW	+	4,568–4,628	61			TCA	59
trnN	+	4,688–4,751	64			GTT	a
rrnS	+	4,752–5,493	742				a
nad1	+	5,494–6,462	969	ATG	TAA		17
trnA	−	6,480–6,542	63			TGC	a
d‐loop		6,543–6,904	362				a
cob	−	6,905–8,110	1,206	ATG	TAA		10
trnT	−	8,121–8,179	59			TGT	1
nad5	+	8,181–9,893	1,713	ATA	TAA		103
trnF	+	9,997–10,056	60			GAA	9
nad4	−	10,066–11,412	1,347	ATG	TAA		−7
nad4L	−	11,406–11,699	294	ATT	TAA		15
trnP	−	11,715–11,775	61			TGG	11
nad6	+	11,787–12,261	475	ATT	T		9
trnS2‐UCN	+	12,271–12,334	64			TGA	a
rrnL	−	12,335–13,515	1,181				a
trnV	−	13,516–13,576	61			TAC	0
trnQ	−	13,577–13,628	52			TTG	101
trnM	+	13,730–13,791	62			CAT	75
nad2	+	13,867–14,865	999	ATA	TAG		−13
trnC	−	14,851–14,902	52			GCA	0
trnY	−	14,903–14,965	63			GTA	

a Gene borders are defined based on borders with adjacent genes.

b Plus strand (+)/minus strand (−).

c Negative values represent overlapping nucleotides.

Furthermore, as found in many mitogenomes, some genes overlap; that is, *atp8*/*atp6*,* nad4L*/*nad4*, and *nad2/trnC* share 4, 7, and 13 nucleotides, respectively, although they are located within different reading frames. Table 2 presents a summary of the organization of the *Bathynomus* sp. mitogenome.

### 3.2 Protein‐coding genes and ribosomal RNAs

With regard to protein‐coding genes, ten (*cox1*–*3*,* atp8*,* atp6*,* nad1*–*3,* and *nad5–6*) are encoded by the plus strand and the remaining three (*cob*,* nad4,* and *nad4L*) the minus strand (Figure 1, Table 2). This orientation is shared by all isopod mitogenomes sequenced to date, except for *nad1*. The 13 protein‐coding genes appear to start with the codon ATN, which is typical for metazoan mitogenomes (Wolstenholme, 1992), and TAN is the termination codon for 11. The *atp8* gene terminates with TGA, which has not been observed in other isopods. Truncated termination codons (T) are observed in *nad6* (Table 2). Posttranscriptional polyadenylation may subsequently generate functional TAA codons (Ojala, Montoya, & Attardi, 1981).

The *rrnL* and *rrnS* genes of *Bathynomus* sp. are 1,181 bp (AT% = 60.5) and 742 bp (AT% = 59.7) in length, respectively. These lengths are typical for crustaceans, whereas the AT contents are slightly lower than those of other isopods (Table 1).

### 3.3 Transfer RNAs

**Figure 2:**
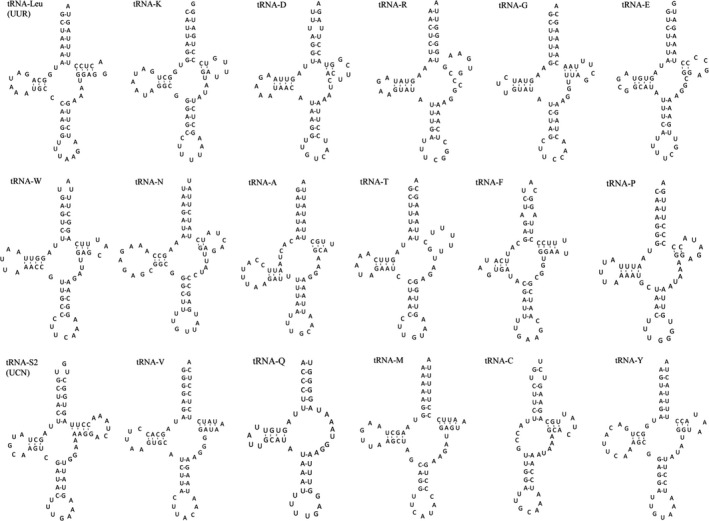
Putative secondary structures for the 18 transfer RNAs of the *Bathynomus* sp. mitogenome. All 18 structures were generated using the MITOS webserver and verified using tRNAscan‐SE and ARWEN. Most tRNAs feature a standard clover‐leaf structure. Exceptions: The TΨC arm is absent from *trnQ*, and the DHU arm is absent from *trnC*

We analyzed the entire genome sequence of *Bathynomus* sp. and successfully identified 18 tRNA genes based on their potential secondary structures (Figure 2) using the MITOS webserver. Moreover, tRNAscan‐SE was used to recheck five genes and ARWEN for 17 genes. The 18 tRNA genes are spread throughout the entire genome and are located on both strands (Figure 2, Table 2). Sixteen of these genes display a common clover‐leaf secondary structure, and the remaining two exhibit a t‐shaped secondary structure.

### 3.4 Genome rearrangement

**Figure 3:**
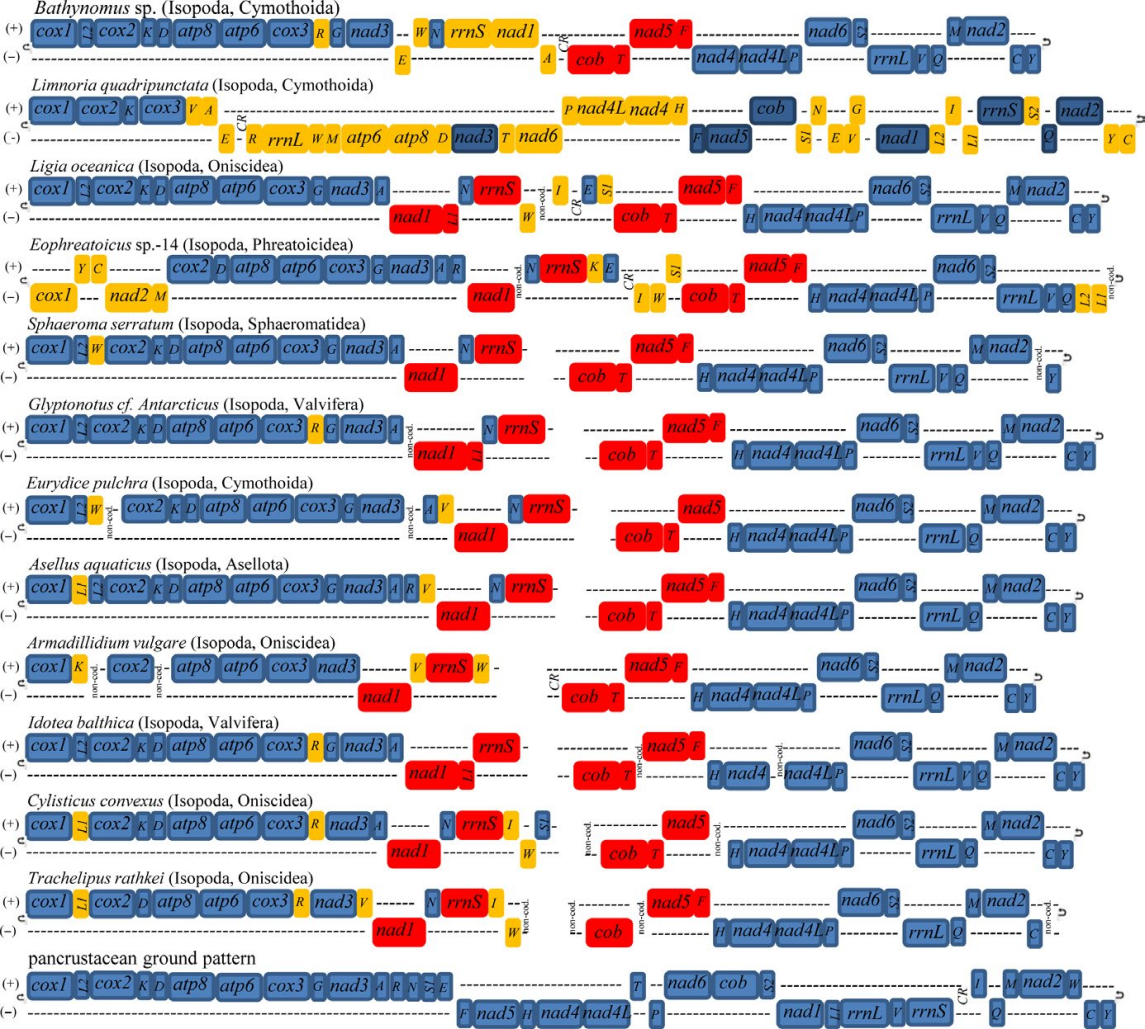
Comparison of mitochondrial gene arrangements in 12 species in the order Isopoda. In addition, the pancrustacean ground pattern (putative ancestral state) is provided. The mt complete genomes of *Bathynomus* sp., *Limnoria quadripunctata*,* Ligia oceanica,* and *Eophreatoicus* sp.‐14 are available; the other mt genomes are incomplete and are missing some tRNAs and the control region (CR). All tRNAs are designated by single letters (except L1, L2, S1, and S2 for *trnL‐CUN*,* trnL‐UUR*,* trnS‐AGN,* and *trnS‐UCN*, respectively). Colored genes denote translocated genes in comparison with the pancrustacean ground pattern. Red indicates genes that share a derived position in isopods. Uniquely derived gene positions of individual species are depicted in yellow

**Figure 4:**
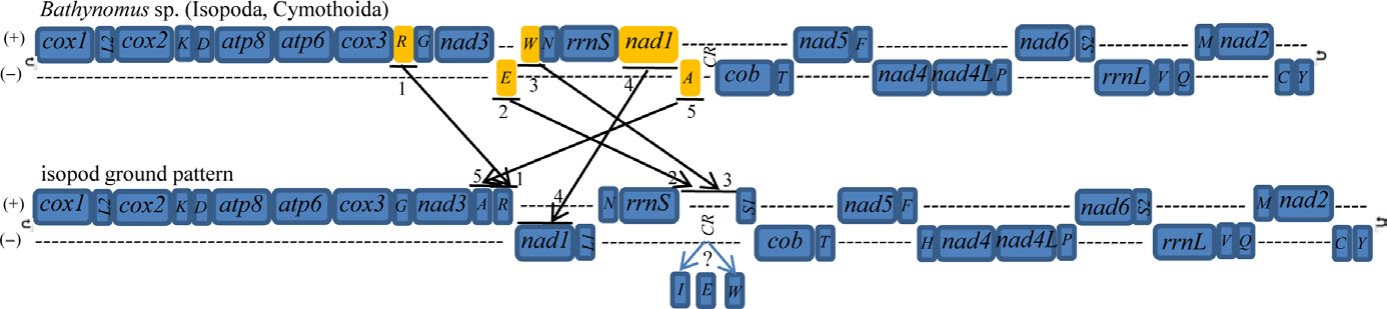
Gene rearrangements of the *Bathynomus* sp. mitogenome. At least five rearrangements have occurred between the *Bathynomus* sp. mitogenome and the putative isopod ground pattern, including (1) *trnR*, (2) *trnE*, (3) *trnW*, (4) *nad1,* and (5) *trnA*. All translocated genes are shown in yellow. All tRNAs are designated by single letters (except L1, L2, S1, and S2 for *trnL‐CUN*,* trnL‐UUR*,* trnS‐AGN,* and *trnS‐UCN*, respectively)

A comparison of all 12 currently available isopod mt genomes, including four complete genomes (Kilpert & Podsiadlowski, 2006, 2010; Lloyd et al., 2015) and eight partial genomes (Chandler, Badawi, Moumen, Greve, & Cordaux, 2015; Kilpert, Held, & Podsiadlowski, 2012; Marcade et al., 2007; Podsiadlowski & Bartolomaeus, 2006), is shown in Figure 3. The putative ancestral state of the pancrustacean ground pattern is also provided (Boore, Lavrov, & Brown, 1998). Between *Bathynomus* sp. and the pancrustacean ground pattern, six tRNA genes rearrangement, one ribosomal RNA gene rearrangement, and three protein‐coding genes rearrangement are shown (Figure 3). However, only four tRNA genes rearrangement and one protein‐coding gene rearrangement are detected between *Bathynomus* sp. and the isopod ground pattern (Figure 4).

### 3.5 Phylogenetic analysis and evolutionary rate estimation

**Figure 5:**
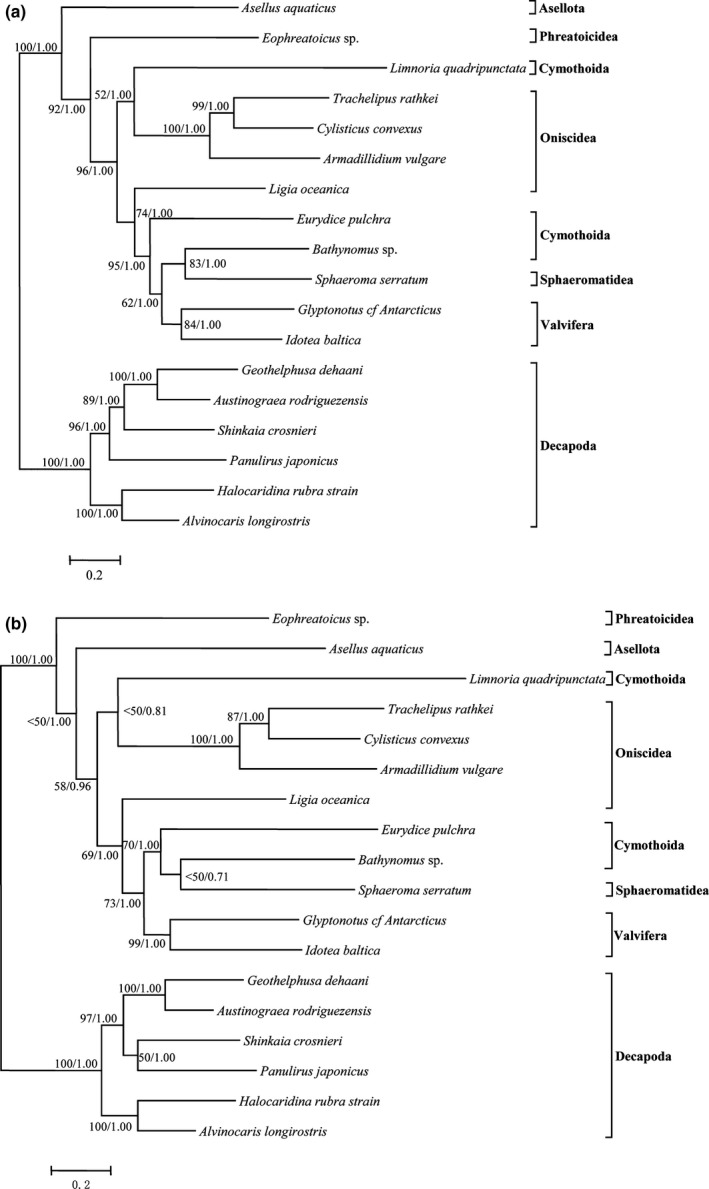
Phylogenetic trees showing relationships among isopods based on nucleotide (a) and amino acid datasets (b). Six decapod species served as outgroups. Branch lengths and topologies are derived from Bayesian analyses. Numbers next to nodes specify bootstrap percentages from the maximum likelihood (ML) analysis plus Bayesian posterior probabilities (BPP). Specifically, <50 indicates bootstrap percentages from ML values below 50%

We examined both the nucleotide and amino acid sequences of protein‐coding genes in intraorder phylogenetic analyses. For each dataset, ML and Bayesian analyses resulted in the same tree topology (Figure 5). The evolutionary rate was estimated using the nucleotide sequences of 13 protein‐coding genes and shown in Table 3. When compared to other suborders, Cymothoida demonstrated the highest evolutionary rate values. However, *Bathynomus* sp. showed the lowest evolutionary rate value in Cymothoida.

**Table 3:** Values for (non)synonymous mutation rates and dN/dS ratios of 13 tandem genes for 18 species. Ancestral branches are not included

Suborder/infraorder	Species	Habitat type	dN^c^	dS^c^	dN/dS^c^
Cymothoida	*Bathynomus* sp.^a^	Deep sea	0.1612	2.2875	0.0705
	*Eurydice pulchra* a	Intertidal zone	0.2002	2.2749	0.0880
	*Limnoria quadripunctata* a	Shallow sea	0.2779	3.2244	0.0862
Oniscidea	*Ligia oceanica* a	Littoral zone	0.1640	2.4777	0.0662
	*Armadillidium vulgare* a	Terrestrial	0.1309	2.9097	0.0450
	*Cylisticus convexus* a	Terrestrial	0.0981	2.5122	0.0390
	*Trachelipus rathkei* a	Terrestrial	0.1265	2.3812	0.0531
Valvifera	*Idotea baltica* a	Shallow sea	0.1335	1.8952	0.0704
	*Glyptonotus cf. Antarcticus* a	Antarctic	0.1344	2.4986	0.0538
Phreatoicidea	*Eophreatoicus* sp. ‐14^a^	Fresh water	0.1910	3.4175	0.0559
Sphaeromatidea	*Sphaeroma serratum* a	Shallow sea	0.1677	2.2522	0.0744
Asellota	*Asellus aquaticus* a	Fresh water	0.2183	3.1446	0.0694
Decapoda	*Shinkaia crosnieri* b	Deep sea vent	0.1128	2.5780	0.0438
	*Panulirus japonicus* b	Shallow sea	0.1375	2.6311	0.0523
	*Halocaridina rubra strain* b	Near the sea shore	0.1212	1.6655	0.0728
	*Geothelphusa dehaani* b	Rivers and streams	0.0876	3.0085	0.0291
	*Austinograea rodriguezensis* b	Deep sea	0.0654	2.4248	0.0270
	*Alvinocaris longirostris* b	Deep sea vent	0.0755	1.7666	0.0427

a 12 species of isopods.

b Six outgroup decapod species.

c *p* < .001.

## 4 Discussion

### 4.1 Transfer RNAs

The DHU arm of *trnC* and the TΨC arm of *trnQ* are completely missing (Figure 2). Loss of the DHU arm from *tRNA‐C* has also been observed in other isopods, such as *Ligia oceanica* (Kilpert & Podsiadlowski, 2006) and *Eophreatoicus* sp.‐14 (Kilpert & Podsiadlowski, 2010). This feature might be a putative autapomorphy of isopoda, as it is absent in other malacostracan crustaceans. In general, tRNAs with a U at the wobble position (the first position) of the anticodon recognize either fourfold degenerate or NNR codons, whereas those with a G at this position only recognize NNY codons (Yang & Yang, 2008). All tRNAs of *Bathynomus* sp. mitogenome obey this rule.

Despite extensive efforts to identify secondary structure in noncoding regions, the genes *trnH*,* trnI*,* trnL1,* and *trnS1* were not found in the mitogenomic sequence. Considering the absence of *trnQ* in the *L. oceanica* mitogenome (Kilpert & Podsiadlowski, 2006), this is not an exception among isopods. Indeed, a lack of these genes is also common in arthropods, such as the absence of *trnQ* in *Aleurodicus dugesii*,* trnS* in *Schizaphis graminum* (Thao, Baumann, & Baumann, 2004), and *trnD* in *Centruroides limpidus* (Davila, Pinero, Bustos, Cevallos, & Davila, 2005). tRNA gene deficiencies have often been observed in protozoans, fungi, algae, plants, and lower metazoans (Schneider & Marechal‐Drouard, 2000); in these cases, imported nuclear DNA‐encoded tRNAs compensate for the lack of mt tRNA. Furthermore, marsupial mt tRNAs exhibit interesting patterns. Janke and Paabo identified in *Didelphis virginiana* a pseudogene similar to *trnD* with an anticodon of GCC instead of the usual GUC (Janke & Paabo, 1993); the authors found that the cytosine is changed to uridine during posttranscriptional RNA editing. Based on its nonfunctional secondary structure, Dorner, Altmann, Paabo, and Morl (2001) discovered a *trnK* pseudogene in the same marsupial species, with compensation due to import of cytoplasmic tRNA rather than RNA editing. As there appears to be no candidate template for RNA editing in the case of *Bathynomus* sp., import of tRNA is a plausible explanation. In addition, some other explanations also may be plausible explanation: (1) specific bias in codon usage. Indeed, a recent work has succeeded in replacing seven codons in *Escherichia coli* (at least part of it), so that the genome can be decoded on 57 triplets rather than 64 (Thiele et al., 2012). It could be that some codons are missing, so that some tRNAs would not be necessary; (2) a hypothesis could be that some of the present tRNA would be edited, so that they would recognize both different amino acids and have different anticodons. This is not entirely impossible, as it is known, in some cases, that the specification of the amino acid loaded on the tRNA uses the anticodon as a signature. In a way, this would require much less genes from the host (just editing) than create a whole RNA translocation across the double membrane of mitochondria.

### 4.2 Genome rearrangement

Compared to the gene orders of other isopod species, the *Bathynomus* sp. mitogenome has undergone significant changes in gene arrangement. First, all 11 isopod mt genomes differ in gene order, but, except for *Limnoria quadripunctata*, most of the differences are limited to the position of one or a few tRNA genes. Second, the gene order of the isopod mt genomes can be clearly distinguished from the pancrustacean ground pattern. Inferred changes in the *Bathynomus* sp. genome involve tRNA genes, protein‐coding genes, rRNA genes, and the mt control region (CR).

Kilpert et al. showed that isopods share a derived order of genes, with individual species experiencing modifications. Using the similarities common to most isopods, a most parsimonious hypothesis for the mt gene order of the isopod ground pattern was inferred, which includes a unique arrangement of *nad1*,* trnL1*,* rrnS*,* CR*,* trnS1*,* cob*,* trnT*,* nad5,* and *trnF* (Kilpert et al., 2012). For *Bathynomus* sp., *cob*,* trnT*,* nad5,* and *trnF* fit the isopod ground pattern, whereas *nad1*,* trnL1,* and *rrnS* strongly diverge from this pattern.

We also found that the mitogenome of *Bathynomus* sp. harbors significant alterations in gene arrangement compared to the gene order of the putative isopod ground pattern described by Kilpert et al. (2012). In total, we identified rearrangements in this species (Figure 4): One rearrangement involves protein‐coding genes, and the remaining are tRNA translocations. Relative to its ancestral position, *trnR* is at a position upstream of *nad3*. *Glyptonotus* cf. *Antarcticus* (Kilpert et al., 2012), *Cylisticus convexus*, and *Trachelipus rathkei* (Chandler et al., 2015) share this rearrangement. The positions of *trnE* and *trnW* are changed to positions downstream of *rrnS*, which has not been found in other isopods to date. Finally, the *nad1* gene is moved to a position downstream of *nad3* and from the minus strand to the plus strand and this translocation is novel feature found only in isopods (Kilpert & Podsiadlowski, 2010; Kilpert et al., 2012; Marcade et al., 2007).

### 4.3 Phylogenetic analysis and evolutionary rate estimation

Indeed, the nucleotide and amino acid trees are nearly the same, except for the position of the Asellota clade (*Asellus aquaticus*) and the Phreatoicidea clade (*Eophreatoicus* sp.‐14). In the case of the nucleotide dataset, the Asellota clade is placed at the base of Isopoda (Figure 5a) and is well supported (ML/BPP = 100/1.00). However, in the amino acid tree, the Phreatoicidea clade is placed at the base of Isopoda (Figure 5b), which is in agreement with the findings of previous research (Kilpert et al., 2012) and is also well supported (ML/BPP = 100/1.00). This discrepancy may be due to the fact that the two clades are represented by only one species each. Neither tree groups *L. quadripunctata* the same clade with the other three Cymothoida species, and *Bathynomus* sp. (Cymothoida) and *S. serratum* (Sphaeromatidea) form one clade in both trees. Kilpert et al. (2012) also found that *Eurydice pulchra* (Cymothoida) and *S. serratum* form a single clade. According to these results, it remains unclear whether Cymothoida is monophyletic or paraphyletic. The other intraorder clade was well reconstructed according to ML and Bayesian analyses of the two dataset (Figure 5). However, as research on the phylogenetic relationships within this infraorder is rare (Kilpert et al., 2012), intensive sampling and analysis of a greater number of species are necessary to determine the phylogenetic relationships among members of Isopoda.

We found that the evolutionary rate of *Bathynomus* sp. is the slowest measured to date within Cymothoida (Table 3), which means that this species may have experienced nonsynonymous mutations harmful to its survival and that its mt genes have been under strong purifying selection (Yang, 2007). This slow evolutionary rate may be associated with the relatively constant deep‐sea environment of this species. For example, *Bathynomus giganteus* was already present as early as 160 million years ago, and a previous study showed that *B. giganteus* specimens from Australia, Mexico, and India are almost exactly the same, possibly because of their nearly identical environments (Parker, 2003). This phenomenon has been found in many high‐altitude habitats species (Shen et al., 2009; Sun et al., 2011).

This study is the first determination of the mitogenome of a deep‐sea member of the order Isopoda, an effort that not only increases sampling of Isopoda but also more importantly provides useful information for understanding the evolution of deep‐sea isopods.

## Conflict of Interest

None declared.
