# Supplementary material for: The first complete mitogenome of the South China deep‐sea giant isopod Bathynomus sp. (Crustacea: Isopoda: Cirolanidae) allows insights into the early mitogenomic evolution of isopods
Source: Ecol Evol. 2017 Feb 16;7(6):1869–81. doi: 10.1002/ece3.2737 (PMC5355201; doi:10.1002/ece3.2737)
Supplement: Supplementary file 1 [file ECE3-7-1869-s001.docx]

**Table S1.** Primers used for determination of the *Bathynomus* sp. mitogenome.

| PCR amplification | Primer name | Primer sequences (5′🡪3′) | Product | Annealing temperature | References |
| --- | --- | --- | --- | --- | --- |
| Partial PCRs  1st round | LCO1490  HCO2198 | GGTCAACAAATCATAAAGATATTGG  TAAACTTCAGGGTGACCAAAAAATCA | 708 bp | 45-48 | Folmer, Black et al. 1994 |
|  | CO2f  CO2r1 | ATTTTTTTYCATGAYCATGC  GGSATTATGTAWGAATCAAATT | 310 bp | 45-48 | In this study |
|  | SR-J14197  SR-N14745 | GTACAYCTACTATGTTACGACTT  GTGCCAGCAGYYGCGGTTANAC | 548 bp | 45-48 | [Simon, Buckley et al. 2006](#_ENREF_3) |
|  | Cytb151F  Cytb270R | TGTGGRGCNACYGTWATYACTAA  AANAGGAARTAYCAYTCNGGYTG | 415 bp | 45-48 | [Merritt, Shi et al. 1998](#_ENREF_3) |
|  | DEnad5F  DEnad5R | TATGTGGDWTWCCTTTTWTAGCDGG  ATHTCAAGMTAARCHAGCHCCHCC | 1001 bp | 45-48 | [Yang and Yang 2008](#_ENREF_4) |
|  | 16sf-cray  16s1472 | GACCGTGCKAAGGTAGCATAATC  AGATAGAAACCAACCTGG | 449 bp | 45-48 | [Crandall and Fitzpatrick 1996](#_ENREF_1) |
| Long PCRs | Cox1-Cox2F  Cox1-Cox2R | CTACCCGCCATTAGCAAGG  TAGAACACGGACCTGAACCC | 1643 bp | 64-68 | In this study |
|  | Cox2-12SF  Cox2-12SR | GCCTGCCTTTGTGCTTATC  AGATTCGCAGCACCTTGAT | 3408 bp | 64-68 | In this study |
|  | 12S-CytbF  12S-CytbR | AACCTGTTGCGTAATCGTCG  GCATCAAACAGGCTCCAATAA | 2305 bp | 64-68 | In this study |
|  | Cytb-ND5F  Cytb-ND5R | AAATGGGAGGAGGAAGTGGA  CTCTTTGAGCCTCCACCACC | 2195 bp | 64-68 | In this study |
|  | ND5-16SF  ND5-16SR | CATTTAGTGGGAGTCAGTGTA  TTACTTTAGGGATAACAGCG | 2425 bp | 64-68 | In this study |
|  | 16S-Cox1F  16S-Cox1R | CCTTCCGTCAGGTCGTTTAT  ATAGCCAAATCAACGGAAGC | 2487 bp | 64-68 | In this study |
| Partial PCRs  2nd round | Cytb1F  Cytb1R | AGTGGGTTATTGGAGCCT  TTGGCGTCACTTTATTCC | 283 bp | 53 | In this study |
|  | Cytb2F  Cytb2R | ATCCAGGTGAGTAGAAGCA  TTCTTCACCCTCCACTTC | 487 bp | 53 | In this study |
|  | 16S1F  16S1R | CCTTCCGTCAGGTCGTTT  GCTAACGAACTCGGCAAA | 148 bp | 55 | In this study |
|  | 16S2F  16S2R | GTCGCTTACCTGCTTGTCG  GCTAACGAACTCGGCAAAC | 472 bp | 55 | In this study |
|  | 61F  61R | CCTGTTGCGTAATCGTCG  GTCTTTCAGGCGAGGGATA | 1131 bp | 54 | In this study |

**
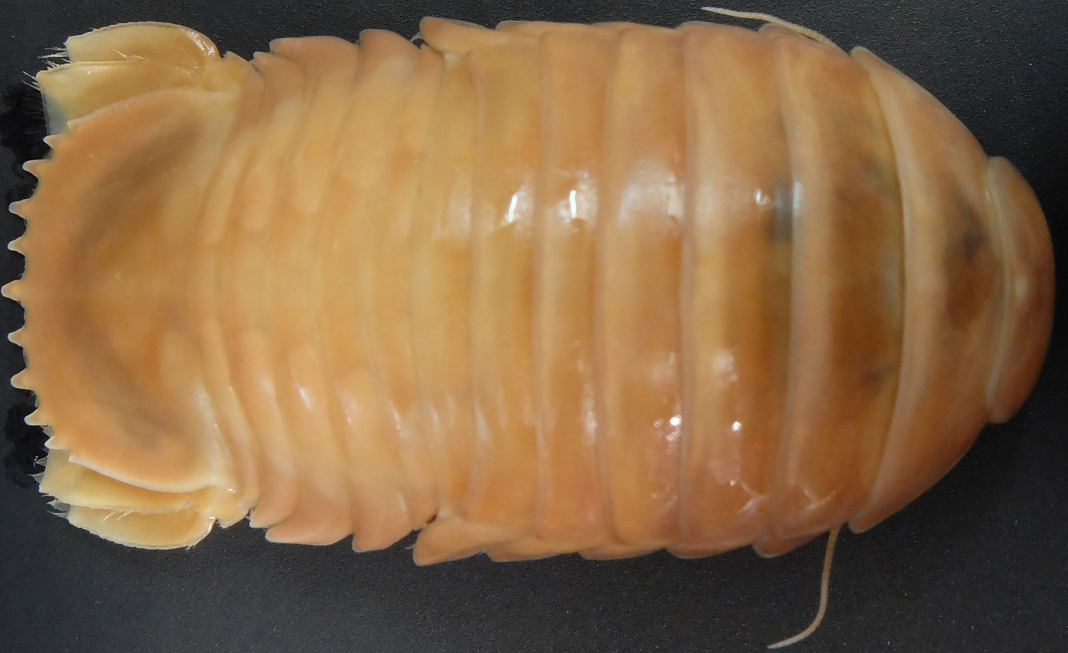
**

**Figure S1.** The study species, *Bathynomus* sp., from the South China deep-sea.
